# Perceptions and experiences of midwives' regarding the use of the labor care guide at two state hospitals in Khomas region, Namibia

**DOI:** 10.1002/ijgo.71184

**Published:** 2026-06-25

**Authors:** Amalia N. N. Ilonga, Takaedza Munangatire

**Affiliations:** ^1^ Faculty of Health Sciences Welwitchia University Windhoek Namibia; ^2^ Department of Nursing University of Namibia Rundu Namibia

**Keywords:** experiences, labor care guide, midwives, perception

## Abstract

The labor care guide (LCG) is a new tool for monitoring labor, introduced to replace the partograph, which had been used improperly and inconsistently across various contexts. Early research suggests that healthcare practitioners view the LCG as a tool that enhances the quality of care during labor and delivery. The aim of this study was to explore midwives' perceptions and experiences of using the LCG at two state hospitals in the Khomas region, Namibia. This study employed a qualitative transcendental (descriptive) phenomenology design. Purposive sampling and maximum variation sampling were applied. A total of 10 midwives' participated in the study. Data were collected through semi‐structured in‐depth interviews and analyzed using thematic analysis grounded in descriptive phenomenology. The study revealed the following four themes; midwives' experiences in transitioning from partographs to LCG in practice, midwives' perceptions of the LCG and adaptation into practice, challenges related to the implementation and use of LCG by midwives' and navigating from incompetence to an expert in using the LCG implication to practice. Despite some negative perceptions, midwives' found the LCG to be a valuable tool in managing labor. However, they recommended appropriate training and some context‐based adjustments to enhance effective utilization.

## 1 INTRODUCTION

The management of labor remains a challenge globally. The partograph was widely used worldwide to detect labor complications early, enabling timely intervention.^1^ For over 40 years, healthcare professionals have used the partograph to monitor labor.^2^ However, the partograph has failed to significantly improve labor care indicators and has faced low utilization rates.^3^ In response, the WHO developed the labor care guide (LCG) in 2018 and has since been adopted in Norway, India, Argentina, Malawi, Zambia, Zimbabwe, Uganda, Kenya, and Tanzania.^4^

The WHO LCG is a paper‐based labor monitoring tool. The first version of the LCG was developed by a WHO expert committee in 2018.^2^ The LCG enables skilled healthcare professionals to provide woman‐centered, safe, and effective care, improving the outcomes and experiences of childbirth for women and babies.^5^ The LCG was officially launched globally in 2020 to facilitate the successful implementation of the WHO's intrapartum care recommendations and to promote high‐quality, evidence‐based, respectful care, regardless of the healthcare setting.^6^ Following the LCG's publication, an evaluation project across six countries assessed its applicability, acceptability, and practicality across different contexts, as well as the barriers and facilitators to its use.^7^, ^8^

This study on the LCG is important in the fight against high maternal and perinatal mortality in sub‐Saharan Africa. Research on the LCG can contribute to achieving the sustainable development goals (SDGs), which aim to reduce global maternal mortality to 70 deaths per 100 000 live births by 2030.^9^ As a relatively new tool endorsed in 2018, studies examining how it has been received and experienced, particularly by midwives' in Namibia, form the basis for further research on the effectiveness of the LCG.

Namibia approved the updated WHO LCG in December 2020. To support the LCG implementation, the Quality Assurance (QA) department was established. Each month, data on LCG completion rates from each hospital are uploaded to the national dashboard. Since the LCG's implementation at two hospitals in the Khomas region, completion rates have ranged from 40% to 65%. This suggests that LCG usage during labor monitoring is not yet consistent. Despite midwives' beginning to use the LCG, their experiences and perceptions of the tool have not yet been studied. Therefore, this study examined midwives' experiences and perceptions following the implementation of the LCG.

## 2 METHODS

### 2.1 Research approach and design

To fully capture the lived experiences of midwives, this study utilized transcendental (descriptive) phenomenology. Unlike other qualitative methods that allow the researcher's interpretation to merge with the data, this approach explicitly requires the researcher to bracket and set aside their personal biases, clinical preconceptions, and institutional assumptions regarding the phenomena under investigation.^10^ This was needed as the researcher herself is a midwife and a frequent user of the LCG. By doing so, the study isolates and focuses entirely on the pure, unfiltered experiences reported directly by the midwives. This methodology was uniquely suited for examining midwives' perceptions and behavioral adaptations during the rollout of the LCG, as its core purpose is to systematically describe, analyze, and comprehend subjective human experiences exactly as they are lived.^11^

### 2.2 Participants and sample

The participants comprised midwives' working in the maternity labor units at two state hospitals in the Khomas region of Namibia who are users of the LCG and provided relevant data necessary to address the research aim. These two state hospitals in the Khomas region are the country's national referral hospitals.

The diverse perceptions and experiences of these midwives' were examined using a non‐probability purposive strategy to gain an in‐depth understanding of midwives' experiences and perceptions regarding the use of the LCG. In descriptive phenomenology, sample size is determined by data saturation rather than statistical representation. Specifically, the eighth interview marked data saturation, and two additional interviews were conducted to confirm the absence of any new viewpoints or ideas. The maximum variation was achieved by purposefully balancing demographics: eight registered and two enrolled midwives, seven females and three males, across an age range of 29–38 and clinical experience of 4–15 years in the maternity ward, including both senior and junior staff.

The study sample size was 10 and was determined by participants who met the inclusion criteria.

The criteria for selecting participants in the interviews were:

- All midwives' working at the two state hospitals maternity wards since the introduction of the LCG. These midwives' have been utilizing the LCG since its launch in December 2020 and have been using the LCG for over two years. The researcher believes they have had adequate exposure to the LCG since its inception and have the necessary data to address the research aims.

The criteria for excluding participants in the interviews were:

- All midwives' working at the two state hospitals maternity wards who had utilized the LCG for less than two years. The researcher excluded these midwives' because they believed they lacked appropriate expertise, particularly during the initial introduction of the LCG into the labor force at both state hospitals, as they were not yet employed at those facilities at the time.

### 2.3 Data collection

The data were collected through semi‐structured, in‐depth, individual interviews, which emphasize the relevance of personal perspective and interpretation, allowing researchers to gain in‐depth insights into the topic under examination. The semi‐structured interview guide was developed by the first author and reviewed by the supervisor and the second author for both face and content validity. The interview guide was developed in English because it is formal and academic in nature and understood by all participants. The questions were developed based on the research aims. In addition, a pilot interview was conducted and analyzed with two participants, which resulted in improved interview skills for the researcher.

The researcher conducted the interviews both telephonically and face‐to‐face in English. Face‐to‐face interviews increased trust and rapport between the interviewer/researcher and the midwives, and telephone interviews allowed participants from diverse geographical locations to connect with the midwives' and were less burdensome for participants than meeting the researcher in person. The researcher informed midwives' about the study's objectives, data confidentiality, and the voluntary nature of participation before conducting interviews. midwives' provided written consent for their interviews to be audio‐recorded. All interviews were conducted in a private room and recorded by the first author, a registered nurse/midwife and a master's student in Nursing Science. Field notes were also taken by the principal investigator after each interview to ensure that midwives' viewpoints were accurately represented. The interviews in this study lasted an average of 30 min. A total of 10 midwives' participated in this study. The researcher collected data continuously, cycling through collection and analysis until no new information emerged. Saturation was identified after the eighth interview, but two additional interviews were conducted to confirm that saturation had been reached. This decision was made because the data variance had leveled off and new perspectives no longer emerged. Additionally, the researcher considered the absence of new insights on the research subject.

These interviews were conducted between July 5, 2023 and October 30, 2023. Following the transcription of the audio recordings, the researcher returned to each hospital and invited participants to review their transcripts to confirm that they accurately reflected their viewpoints.

### 2.4 Data analysis

Data were analyzed using thematic analysis based on descriptive phenomenological using Sundler et al.^12^ three steps as a guide. This allowed the researcher to comprehend patterns of meaning from data on actual experiences. Step 1: Achieve familiarity with the data through open reading, whereby the researcher read over each interview transcript four times to gain a sense of what the data was revealing. While reviewing the data, the researcher began to investigate the perceptions and experiences expressed in the transcripts, particularly evaluating how midwives' perceptions and experiences with the LCG were narrated and interpreted throughout the interviews. Step 2: Search for meanings and themes: Parts of the data were further illuminated, deepening the search for meaning and themes. While reading the interview transcripts, meanings relevant to the study's aim were highlighted. Step 3: Organize themes into a meaningful wholeness: The researcher then reviewed the meanings of lived experiences, identified patterns, and began developing themes. The researcher continued to examine the patterns and, depending on their similarities and differences, fitted them into themes. Furthermore, the researcher highlighted all the meanings of lived experiences and sentences that matched these phrases, adding new meanings as the researcher read the rest of the interview text. After reviewing all the interview texts, the researcher organized the data into groups, defined as themes. Coding and themes were first developed independently by the first author and then checked by the research supervisor for agreement.

### 2.5 Rigor and trustworthiness of the study

Trustworthiness in this study served as evidence for the claim that the investigation's conclusions warrant attention.^13^ Trustworthiness in this qualitative research was achieved by ensuring credibility, transferability, dependability, and confirmability.^14^ The credibility of the results was strengthened through member checking, during which the transcripts were returned to the participants for confirmation. The researcher, who has also used the LCG, applied reflexivity by maintaining an open‐minded attitude, recognizing personal biases, and exercising self‐reflection throughout the study process. External audits and peer check improved dependability and conformability. The research supervisor, who has published numerous qualitative research papers, reviewed and revised the transcripts, codes, and categories to identify any coding issues. Also, the researcher maintained a record of decisions made during the study, including changes in techniques and analyses, thereby improving transparency and accountability. This audit trail helped establish the study's reliability and provides insights into potential biases. Lastly, transferability was addressed through verification of the study results by two midwives' who did not take part in this study but work in the maternity antenatal ward and use the LCG; they found resonance in the findings.

### 2.6 Ethical considerations

The midwives' were fully informed about the study's specific objectives, the voluntary nature of participation, and the assurance of data confidentiality prior to the interviews. They then provided written informed consent for participation and audio recording in private rooms. This research was guided by three ethical principles: respect for persons, beneficence and non‐maleficence, and justice.

## 3 RESULTS

### 3.1 Participant characteristics

A total of 10 midwives' were interviewed for this study. The data analysis revealed that midwives' were aged 29–38 years. A total of seven (70%) of the participants were female, while three (30%) were male. A total of eight (80%) of the participants were registered midwives, while two (20%) were enrolled midwives. The midwives' work experience ranged from 4 to 15 years; however, they all confirmed having 2.5 years of experience using the LCG in their antenatal ward, corresponding to the same period since its introduction. The results of the thematic phenomenological descriptive analysis yielded four themes.

### 3.2 Theme 1: Midwives' experiences in transitioning from partographs to the LCG in practice

The transition from the partograph to the LCG involves a cyclical adaptation process requiring expert training and mentorship. The initial reactions and clinical adjustments of midwives' were heavily shaped by the structural and design differences between the two tools.

Midwives' initially anticipated that the LCG would be more challenging due to its text‐heavy nature, contrasting with the familiar, highly graphic layout of the partograph.

When we started using the labor care guide, it seemed like this is a difficult tool because there were just numbers, unlike the partograph where it's even showing you that there's an alert line, there's an action line and so forth. [P2]

Midwives' responded positively to the structural changes after implementation. The LCG allows for more thorough documentation of assessments and plans, and consolidates extensive information onto a single page, making clinical review faster and more efficient.

-
  Expanded documentation: “What I like about the labor care guide is the fact that it gives us space or place to enter all our assessments and our plans. Because I noticed that the partograph didn't give us that.” [P7]
-
  Efficiency: “You can just look at the labor care guide once and you have all data that could probably take around 3 to 4 pages if it has to be enumerated in any other manner…” [P9]

One key benefit highlighted was the addition of specific maternal monitoring elements that the partograph lacks. This change in structure directly promotes supportive, woman‐centered care by enabling the routine assessment of hydration, pain management, and the presence of a birth companion.

-
  Companionship integration: “…the labor care guide is even allowing women to have a companion which was not in the old partograph.” [P2]
-
  Structured support: “A woman, at least, should have a companion during labor… So now when you are filling this up, you tend to ask the mummy if she has a companion that she wishes to have.” [P4]

On the other hand, midwives' found that the partograph and the LCG are comparable in the way they monitor women in labor. As a result, they experienced an easier adjustment to using the LCG because the monitoring frequency remained consistent.

“The contractions are still monitored the same, half‐hourly, including the fetal heart rate.” [P5]

### 3.3 Theme 2: Midwives' perceptions of the LCG and adaptation into practice

This theme focuses on midwives' perceptions of the LCG and their adaptation to using it.

Midwives' observed that the LCG helps curb unnecessary medical interventions. Replacing the fixed alert and action lines of the traditional partograph with flexible, time‐based threshold guidelines supports safe, evidence‐informed clinical practice.

-
  Delayed initiation and better outcomes: “Yes, it's going to improve the birth outcome than the partograph because a partograph is opened way early.” [P9]
-
  Preventing over‐intervention: “…unnecessary interventions were prevented because now it's guiding us on how to wait. It gives us the duration of time a woman can be at a certain dilatation.” [P4]
-
  Professional growth: “It was very exciting actually. It's always exciting to learn new things and the way it was in detail…” [P1]

Moreover, the design of the assessment components of the LCG promotes critical thinking rather than rote tracking, stimulating midwives' to develop customized care plans and to engage in collaborative decision making with the mother.

-
  Comprehensive support: “Each patient will require you to kind of like apply the labor care guide differently somehow. It helps me to comprehensively… assist the patient…” [P6]
-
  Collaborative practice: “In all that, they added also shared decision making whereby we as nurses, can also monitor and see what decisions we can make.” [P3]

Initial resistance was very common among frontline personnel. The main difficulty was the loss of the familiar graphical effect of the partograph, which first made it harder for midwives' to mentally picture labor progress.

-
  Systemic resistance: “Like any change is always accepted with mixed feelings. Whereby people high up the hierarchy… will embrace it. And while people were a bit low… will usually repel a change with no rational…” [P9]
-
  Loss of graphical plotting: “It was kind of a weird care guide because normally the first partograph… was very for me much easier. We would just plot the graphs according to the lines.” [P3]
-
  Tracking difficulty: “…the graph used to give us a clear indication on how the progress of labor would go.” [P7]

Later, midwives' effectively adapted despite early implementation difficulty and anxiety of change. Completion rates leveled off over time, and staff reported high levels of comfort using the tool to identify obstetric warning indicators and to work with doctors.

-
  Overcoming friction: “Even though there was a bit of discrepancy in the beginning, people have adjusted to it because of the fear of change, but the labor care guide now is being completed.” [P7]
-
  Clinical confidence: “Now we understand the full capacity, we understand how to use it, and we also know actually like warning signs… and then we know when to also notify the doctors.” [P3]

### 3.4 Theme 3: Challenges related to the implementation and use of LCG by midwives

Transitioning to the LCG requires shifting from a hierarchical to an independent clinical mindset. Frontline midwives' identified critical tool‐design, resource, and systemic challenges that hinder effective implementation.

First, midwives' identified structural flaws in the LCG design that create space constraints, making it difficult to promptly spot clinical problems and hindering accurate data capture.

-
  Inaccurate column segmentation: “Some columns represent only one hour and when you are doing the fetal heart rate and the pulse of the mother it's a challenge as they are supposed to be done every 30 minutes.” [P2]
-
  Poor visibility and restricted space: “It's not also easier to see abnormalities such as maternal tachy. You would have to shrink your pupils to see what exactly the number is like… we wish it could be more like the graph, at least on an A3.” [P3]

It was further reported that the LCG requires rigid monitoring frequencies, which are directly compromised by systemic limitations in medical equipment and manpower. This results in the tool being left incomplete during high‐volume shifts.

-
  Equipment deficits: “We don't have a lot of CTGs also in our wards, so that would also cause the delay in fetal heart rate monitoring…” [P3]
-
  Manpower shortages: “…sometimes during busy times, the labor care guides sometimes are left not complete, due maybe to that day it's a busy day.” [P10]

The LCG encourages labor companions to promote women‐centred care, but the current hospital infrastructure is not physically intended to accommodate them, resulting in severe privacy violations.

-
  Privacy conflicts: “…it's a challenge because of privacy you can't have both companions for both women in the same room.” [P6]
-
  Inadequate layout: “The facility actually is not built in that in that capacity where we have to include the companion.” [P8]

A major problem in implementing the LCG was a lack of training and ongoing support. While the LCG user's manual highlights irregularities, it does not specify what midwives' should do if they encounter an alert during labor monitoring.

-
  Lack of clinical guidance in user's manuals: “The labor care guide manual doesn't emphasize on what you should do if you come across an alert line so there, you just have to use your brain or inform the doctor.” [P5]/“The labor care guideline doesn't show us how to manage any abnormalities…” [P3]
-
  Absent training rollout: “We just received files with this newly revised labor care guidelines without training, so we just have to start using it.” [P8]
-
  No post‐implementation support: “Now we're picking up gaps and questions and things that don't make sense… You wish to go back and ask and there's just no one to ask again.” [P4]

### 3.5 Theme 4: Navigating from incompetence to an expert in using the LCG. Implication to practice

Becoming proficient with the LCG is a slow and largely unsupported process. Without consistent, formalized institutional training, midwives' depend primarily on self‐directed learning and informal peer support to fill significant knowledge gaps.

-
  Self‐directed online learning: “The third way I learned was through YouTube. I just googled how to use the labor care guide and that's how I got to.” [P4]
-
  Incomplete understanding: “We just decided to go ahead with what we have observed on YouTube, and what we have read in the manual all the time, but we don't understand exactly everything.” [P4]

Likewise, although peer discussions and shadowing offer important support, heavy workloads often undermine these informal mentoring arrangements, leaving newly appointed staff to navigate the tool without guidance.

-
  Peer knowledge sharing: “We had some discussion with colleagues regarding the labor care guide, that's how I knew how to use.” [P5]
-
  Intended mentorship design: “They are allocated to mentors. The senior staff are the ones that get to accept them in the system… familiarizing them with the… labor care guide…” [P6]
-
  Systemic breakdown due to high volume: “…mostly for new staff we get to shadow them. But even though there's a high number of patients sometimes. The new staff don't really get to be mentored.” [P6]

Consequently, previous formal training efforts are remembered as either long past or lacking in depth. Simply attending a session did not translate into clinical competence, leaving staff to contend with persistent practical difficulties on the ground.

-
  Outdated onboarding: “Our unit was offered training, but that was also a long time ago. It was like 2 years ago if I can remember well.” [P4]
-
  Superficial training content: “…we didn't get proper training… it was just like, ok, this is what we have so we have to start using it. I think it was not adequate enough because we still got some few challenges after the training.” [P10]

Midwives' are resistant to standalone, theory‐heavy workshop formats. Having gained practical experience with the tool, they are calling for hands‐on refresher training conducted at the ward level, where they can bring specific, experience‐informed questions directly to clinical experts.

-
  Absence of dedicated trainers: “Help from organizers of the labor care guide to teach midwives' on the floor that one we don't have, we just ask as colleagues for help.” [P10]
  “We need training. Training so everybody can understand.” [P5]
-
  Preference for ward‐based, practical delivery: “…it can be a hands‐on training, which is just not really it doesn't need to be like workshops… it can just be as refreshers in the ward. And it can be for students and as well as the midwives' on the floor.” [P7]
-
  Proactive demand for post‐exposure upskilling: “We have approached the unit manager and the matron… we wish to have another mentor or a clinical instructor to come teach us again because we are awake now. We know what we are facing. We know what we can ask unlike when you are informing the person for the first time…” [P4]

## 4 DISCUSSION

The findings of this study provide important insights into the real‐world implementation of the WHO's LCG in the Namibian public health sector and offer a useful comparative perspective on wider sub‐Saharan African experiences. This discussion explores the lived experiences of midwives' at two national referral hospitals in the Khomas region to determine how localized systemic factors, behavioral modifications and informal learning structures compare with or differ from multicountry African cohorts, namely the landmark assessments of Vogel et al.^8^

In general, implementing the LCG requires meeting standards, including adequate facilities, appropriate equipment, and qualified providers who possess critical thinking, analytical and synthetic skills, independent decision making abilities, and teamwork skills. Therefore, an appropriate delivery room system setup is necessary, encompassing three key elements: hardware, software/standard operating procedures (SOPs), and the provider's ability to work both independently and collaboratively as part of a team.

### 4.1 Graphic monitoring and the resistance to change

A major behavioral element that emerged was the initial reluctance to the shift from the classic partograph to the LCG. Midwives' in this research were somewhat resistant; they said that the LCG was more difficult structurally, since it required extensive numerical entry rather than the familiar graphical style of the partograph. This result reflects African literature across multiple countries. Laisser et al.^4^ reported that midwives' from multiple regional sites were greatly surprised and initially unhappy when they heard that the partograph would be phased out; they claimed they were perfectly happy with the partograph and saw no legitimate reason for change.

This resistance points to a deep cross‐border systemic pattern. LCG users in low‐resource settings become strongly dependent on the graphic prompt impact of the partograph alert and action lines. The Namibian midwives' argued that the partograph provides a more transparent, immediate indication of irregular labor patterns. African cohorts have confirmed this sentiment, finding that without alert lines, clinical decisions are harder to make, necessitating a far greater level of independent analytical knowledge from providers. Nevertheless, the Namibian midwives' demonstrated a robust ability to adapt to the new tool and, despite initial barriers and “fear of change” reported, it was eventually accepted and completed regularly as they became more comfortable through practice. This is in line with the multicountry study by Vogel et al.,^8^ who found that although early resistance is created by the introduction of a non‐graphical tool, persistent clinical exposure eventually leads to stable user compliance.

### 4.2 Resolving shared African structural challenges

The Namibian midwives' perceptions and experience with the LCG demonstrate the same structural and physical inadequacies documented across sub‐Saharan Africa in diagnosing the operational limitations of the LCG. Namibian midwives' strongly criticized the short columns, restricted data input boxes and micro‐font sizes of the standard LCG sheet since they caused visual strain and increased the likelihood of data entry mistakes. This structural issue is the same as the problems observed by Laisser et al.,^4^ in which African participants indicated that an insufficient number of boxes and a small font were key obstacles to use. To optimize usability, future generations of the LCG sheet should be physically enlarged, potentially using a single, complete large‐page style identical to the intensive care unit monitoring charts to conveniently incorporate long‐form clinical notes.

The real implementation of the LCG is currently being hindered by serious resource and infrastructural limitations outside borders, beyond paper design:

- Equipment constraints: Namibian midwives' reported persistent shortages of cardiotocograph (CTG) devices, which directly delayed or undermined the required 30‐min intervals for fetal heart rate (FHR) monitoring. This is similar to the broader study across Africa by Vogel et al.,^8^ who found that doctors in countries such as Kenya, Malawi, Nigeria, and Tanzania continued to struggle to obtain basic diagnostic supplies, such as Doppler monitors.
- Staffing shortages: The emergence of serious staffing shortages during peak clinical hours at Khomas hospitals meant that the extensive LCG was often not completed. This is highly consistent with East African statistics from Kenya, which found that a single midwife is often required to deal with up to 10 women in labor at the same time, making the detailed and continuous documentation required by the LCG almost difficult Vogel et al.^8^
- Infrastructural layouts: One of the primary innovations of the LCG is the official tracking of support for mother care, such as labor companionship. However, for Namibian midwives, this component was found to be quite difficult as public hospital wards were physically structured without providing for individual privacy or room for companions. This operational mismatch is clearly reinforced by Vogel et al.,^8^ who establish that across sub‐Saharan Africa, standard delivery rooms are fundamentally unequipped to accommodate companions without compromising the privacy of nearby patients.

Consequently, the measurement of the LCG's genuine clinical effectiveness in lowering maternal and perinatal mortality will remain highly compromised across Africa unless greater macro‐level shortcomings in staffing, equipment supply and hospital architecture are addressed by institutional stakeholders.

### 4.3 The digital pivot: An innovative contribution to LCG implementation

This study contributes to the international LCG literature by capturing how midwives' transition from novice to expert without official institutional support. A significant clinical gap identified in Namibia was that many midwives' had to implement the revised LCG overnight with no prior formal training, and there was a total lack of designated clinical instructors to answer operational questions on the floor.

Faced with this institutional vacuum, the Namibian midwives' overcame established structures and created a route to informal, self‐directed digital learning networks, using open‐access internet platforms such as YouTube to learn how to record labor progress and interpret the tool. While research by Pandey et al.^15^ recognizes the benefits of online training videos for enhancing international LCG acceptability, this study empirically demonstrates that midwives' are already using these platforms as frontline coping strategies.

While this digital innovation offers significant advancements, it simultaneously introduces distinct challenges. The participants themselves disclosed that relying exclusively on unverified YouTube videos and self‐interpreted instructions did not always lead to an accurate or thorough understanding of the full potential of the LCG. In this high‐risk obstetric setting, the unregulated and fast transmission of incorrect or unconfirmed digital clinical material might dangerously promote poor labor monitoring techniques. Therefore, informal peer cooperation and workplace mentorship remain essential, but they cannot substitute for the urgent need for organized, hands‐on refresher training directly in the clinical wards. Institutional organizers need to formalize these digital and peer frameworks so that the learning curve from beginner to expert is paved with verifiable, evidence‐based instructions rather than random online searches.

### 4.4 Limitations

Given the narrow scope of this study to the economically stable and better‐funded capital region (Khomas), the authors strongly suggest a larger, more nationally representative field study across the under‐resourced rural regions of Namibia to assess the effectiveness of large‐scale LCG. Limitations of interviews include the researcher's influence on the data, such as biases, beliefs, and values introduced into the study from the outset. Throughout the study, the researchers had to maintain an open‐minded attitude, recognize their personal biases, and engage in self‐reflection to ensure their credibility. Finally, another limitation was that individuals opted out of the study because they were not comfortable with how data was collected through interviews, forcing the researcher to search for additional participants.

## 5 CONCLUSION

While midwives' successfully transitioned from novices to experts, measuring the true medical effectiveness of the LCG is impossible if systemic failures such as unaccommodating ward infrastructure for companions, severe manpower shortages during peak hours, and deficient guidelines in the user manual are left unaddressed by hospital management. All this should be overcome.

## AUTHOR CONTRIBUTIONS

Amalia N. N. Ilonga: Substantial contributions to the conception, design of the work and the acquisition, analysis, and interpretation of data for the work; drafting the work and reviewing it critically for important intellectual content; final approval of the version to be published; agreement to be accountable for all aspects of the work in ensuring that questions related to the accuracy or integrity of any part of the work are appropriately investigated and resolved. Takaedza Munangatire: Substantial contributions to the conception, design of the work and the analysis and interpretation of data of the work; reviewing the work critically for important intellectual content; final approval of the version to be published; agreement to be accountable for all aspects of the work in ensuring that questions related to the accuracy or integrity of any part of the work are appropriately investigated and resolved.

## FUNDING INFORMATION

The authors have nothing to report.

## CONFLICT OF INTEREST STATEMENT

The authors have no conflicts of interest.

## DISCLOSURE

AI was not used in the preparation of this work.

## Data Availability

Research data are not shared.
